# Autophagy activated by tuberin/mTOR/p70S6K suppression is a protective mechanism against local anaesthetics neurotoxicity

**DOI:** 10.1111/jcmm.13003

**Published:** 2016-11-15

**Authors:** Jingwei Xiong, Qiuyue Kong, Leyang Dai, He Ma, Xiaofei Cao, Li Liu, Zhengnian Ding

**Affiliations:** ^1^ Department of AnesthesiologyFirst Affiliated Hospital with Nanjing Medical UniversityNanjingChina; ^2^ Department of GeriatricsFirst Affiliated Hospital with Nanjing Medical UniversityNanjingChina

**Keywords:** local anaesthetics, neurotoxicity, autophagy, tuberin/mTOR/p70S6K signalling

## Abstract

The local anaesthetics (LAs) are widely used for peripheral nerve blocks, epidural anaesthesia, spinal anaesthesia and pain management. However, exposure to LAs for long duration or at high dosage can provoke potential neuronal damages. Autophagy is an intracellular bulk degradation process for proteins and organelles. However, both the effects of LAs on autophagy in neuronal cells and the effects of autophagy on LAs neurotoxicity are not clear. To answer these questions, both lipid LAs (procaine and tetracaine) and amide LAs (bupivacaine, lidocaine and ropivacaine) were administrated to human neuroblastoma SH‐SY5Y cells. Neurotoxicity was evaluated by MTT assay, morphological alterations and median death dosage. Autophagic flux was estimated by autolysosome formation (dual fluorescence LC3 assay), LC3‐II generation and p62 protein degradation (immunoblotting). Signalling alterations were examined by immunoblotting analysis. Inhibition of autophagy was achieved by transfection with beclin‐1 siRNA. We observed that LAs decreased cell viability in a dose‐dependent manner. The neurotoxicity of LAs was tetracaine > bupivacaine > ropivacaine > procaine > lidocaine. LAs increased autophagic flux, as reflected by increases in autolysosome formation and LC3‐II generation, and decrease in p62 levels. Moreover, LAs inhibited tuberin/mTOR/p70S6K signalling, a negative regulator of autophagy activation. Most importantly, autophagy inhibition by beclin‐1 knockdown exacerbated the LAs‐provoked cell damage. Our data suggest that autophagic flux was up‐regulated by LAs through inhibition of tuberin/mTOR/p70S6K signalling, and autophagy activation served as a protective mechanism against LAs neurotoxicity. Therefore, autophagy manipulation could be an alternative therapeutic intervention to prevent LAs‐induced neuronal damage.

## Introduction

Local anaesthetics (LAs) are widely used for peripheral nerve blocks, epidural anaesthesia, spinal anaesthesia and pain management. However, epidemiological studies demonstrate that high dosage and long duration of exposure of LAs could provoke potential neuronal toxicity, as manifested by transient neurologic syndrome, cauda equina syndrome and delayed sacral nerve disorder 1, 2, 3, 4, 5, 6. Most damages are transient and often subclinical or present as mild mononeuropathies, however, major complications could be also resulted from permanent neuron damage 1, 2. Therefore, it is important to identify the intrinsic protective mechanism for the prevention of LAs neurotoxicity.

Autophagy is an evolutionarily conserved process which sequesters cytoplasmic materials for lysosome‐dependent degradation 7, 8, 9. Under normal conditions, autophagy is important for maintaining cellular homeostasis by turning over damaged materials 10. Autophagy is activated by a wide spectrum of cellular stresses 10, 11, however, autophagy activation could be either beneficial or harmful to cells under pathological conditions depending on the cell types and stimuli 12, 13.

Evidence has shown that autophagosome formation is negatively regulated by mTOR/p70S6K signalling pathway 7, 14, 15. The mTOR is activated by functional inactivation of tuberin after its phosphorylation by Akt 16, 17. Intriguingly, we have reported that bupivacaine suppressed Akt activation in neuronal cells 18, 19, 20, suggesting a possible role of LAs in activation of autophagosome formation. However, it remains unknown about how does LAs affect autophagy process in neuronal cells and what role does autophagy play in LAs neurotoxicity.

To address these questions, we treated human neuroblastoma SH‐SY5Y cells with both lipid LAs (procaine and tetracaine) and amide LAs (bupivacaine, lidocaine and ropivacaine) at previously described dosages 18, 19, 21, 22, 23, 24. The results showed that all the examined LAs up‐regulated autophagic process and inhibited tuberin/mTOR/p70S6K signalling in neuronal cells. Moreover, inhibition of autophagy aggravated the LAs‐provoked neurotoxicity. Collectively, the data suggest that autophagy activation is a protective mechanism against LAs neurotoxicity. Manipulation of autophagy could be an alternative approach for preventing LAs‐induced neuronal damage.

## Materials and methods

### Chemicals and antibodies

Bupivacaine, lidocaine, procaine, tetracaine and primary antibody for α‐Tubulin were purchased from Sigma‐Aldrich (St. Louis, MO, USA). Ropivacaine was from Meilun Bioteck (Dalian, China). Primary antibodies for LC3, p62, beclin‐1, mTOR, phospho‐mTOR (p‐mTOR), p70S6K and phospho‐p70S6K (p‐p70S6K), tuberin and phosphor‐tuberin (p‐tuberin) were from Cell Signaling (Beverly, MA, USA). Bafilomycin A1 was from Calbiochem (San Diego, CA, USA). MTT [3‐(4,5‐dimethylthiazol‐2‐yl)‐2,5‐diphenyltetrazolium bromide] reagent was from Bio Besic, Inc (Markham, ON, Canada). Lipofectamine 2000^®^ reagent was from Life Technologies. BCA protein assay kit and supersignal west pico chemiluminescent substrate were obtained from Pierce (Rockford, IL, USA). siRNA of beclin‐1 was synthesized by GenePharma (Shanghai, China).

### Cell culture

Human neuroblastoma SH‐SY5Y cells were obtained from ATCC and were grown in DMEM containing 10% foetal bovine serum, 100 U/ml penicillin and 100 μg/ml streptomycin at 37°C with 5% CO_2_.

### Mouse primary neuron culture and Las treatment

Primary cortical neurons were isolated from the brains of newborn mice (<3 days) as described previously 25, 26. Briefly, the brain cortex was dissociated by incubating with trypsin/ethylenediaminetetraacetic acid (EDTA), and then plated onto ECM Gel‐coated culture dishes in Neurobasal^™^ medium containing 2% B27 supplement. After growth for 7 days, neurons were challenged with tetracaine and bupivacaine for 6 hrs. After then cells were collected for immunoblotting analysis.

### MTT assay

The MTT assay was used to evaluate cell viability according to previous methods 18, 19. Briefly, cells that grown in 24‐well plates were stimulated with LAs for 24 hrs at indicated concentrations. After then the cells were incubated with MTT (0.5 mg/ml) at 37°C for 4 hrs. The formed crystals were solubilized in 100 μl of DMSO and the colour was read photometrically at 570 nm on Synergy HT plate reader (Bio‐Tek Inc., Winooski, VT, USA). The percentage of survived cells over controls was calculated. The median death dose (LD50) was calculated.

### Morphological study

Cells that grown in 24‐well plates were stimulated with LAs for 24 hrs at indicated concentrations. After then, cell morphology was examined by phase‐contrast light microscope (Axiovert 200; Zeiss Ltd., Gottingen, Germany) at a magnification of 400×. Three images were taken randomly in each well.

### Dual fluorescence‐based LC3 punctuation assay

SH‐SY5Y cells were transiently transfected with a dual fluorescent (mRFP‐EGFP) ptfLC3 plasmid (Plasmid #21074; Addgene, Cambridge,MA.) 27. Twenty‐four hours after transfection, LAs were administrated to cells for 6 hrs. Cells treated with starvation, which was induced by incubation with Hank's buffer containing 0.1% BSA, served as autophagy‐positive controls. Bafilomycin A1‐treated cells served as autophagy‐negative controls. The fluorescence images were observed with a confocal microscope. Autolysosomes and autophagosomes were counted and percentages of autolysosomes were calculated.

### Western blot analysis

Western blotting was performed as described previously 18, 19. Cells that grown in 60‐mm dishes were challenged with LAs for 6 hrs. Cell lysates were subsequently prepared using lysis buffer (50 mM Tris, pH 7.4, 150 mM NaCl, 0.5 mM EDTA, 0.5% sodium deoxycholate, 0.1% SDS and 1% NP‐40). After centrifuged for 10 min at 7900 × g, the supernatants were subjected to protein assay. Equal amount of proteins (30 μg) was separated by SDS‐polyacrylamide gels and transferred onto Immobilon‐P membranes (Millipore Corp., Bedford, MA, USA). After blocking with 5% fat‐free milk, the membrane was incubated with the appropriate primary antibody overnight at 4°C, followed by the appropriate secondary antibody. The blots against anti‐α‐tubulin served as loading controls. Signals were detected with an ECL kit and quantified by scanning densitometry.

### siRNA transfection

SH‐SY5Y cells were transfected with beclin‐1 siRNA (sense 5′‐3′ GCUGCCGUUAUACUGUUCUTT and antisense 5′‐3′ AGAACAGUAUAACGGCAGCTT) with Lipofectamine 2000^®^ reagent according to the previous studies 7, 28. Cells transfected with scrambled RNA served as negative controls. The beclin‐1 knockdown efficiency was evaluated by immunoblotting analysis 48 hrs after transfection. Forty‐eight hours after transfection, cells were exposed to LAs for indicated duration.

### Statistical analysis

Data are presented as mean ± S.D. Groups were compared using Student's two‐tailed unpaired *t*‐test or one‐way anova analysis followed by Tukey post hoc test, as appropriate with SPSS 13.0 software (SPSS Inc., Chicago, IL, USA). Statistical significance was set at *P <* 0.05.

## Results

### LAs decreases cell viability in a dose‐dependent manner

**Figure 1:**
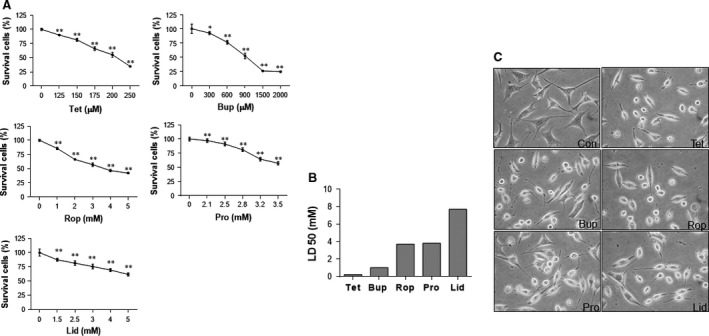
Neurotoxicity of LAs in SH‐SY5Y cells. (**A**) Dose–effects relationship. Cells were challenged with tetracaine, bupivacaine, ropivacaine, procaine, lidocaine for 24 hrs at the indicated concentrations. Cell viability was evaluated by MTT assay. **P < 0.01 and *P < 0.05 *versus* untreated controls. *n* = 3–6 per group. (**B**) LD50 (Median lethal dose). The LD50 was calculated based on the measurement of dose effects of LAs on cell death in **A**. The neurotoxicity of LAs was tetracaine > bupivacaine > ropivacaine > procaine > lidocaine. *n* = 3–6 per group. (**C**) Cellular morphology. Cells were treated with LAs for 24 hrs. Cell morphology was observed under a phase‐contrast microscope at a magnification of 400×. Representative images from three independent experiments are shown. Con: control; Tet: tetracaine; Bup: bupivacaine; Rop: ropivacaine; Pro: procaine; Lid: lidocaine.

SH‐SY5Y cells were treated with bupivacaine, lidocaine, tetracaine, procaine and ropivacaine at different dosages according to the previous studies 7, 18, 19, 21, 22, 23, 24. Cell viability was evaluated by MTT assay 24 hrs after LAs challenge. As shown in Figure 1A, tetracaine significantly decreased viability by 9.9, 18.3, 33.9, 44.8 and 65.1% at the dosages of 125, 150, 175, 200 and 250 μM, respectively, compared with the untreated controls (*P* < 0.01). Similarly, dose‐dependent decreases in viability were detected in the cells treated with bupivacaine (300–2000 μM), ropivacaine (1–5 mM), procaine (2.1–3.5 mM) and lidocaine (1.5–5 mM), respectively, compared with untreated controls (*P* < 0.01 or 0.05).

### LD50 of LAs

The LD50 was 212.7 μM for tetracaine, 989.1 μM for bupivacaine, 3.6 mM for ropivacaine, 3.7 mM for procaine and 7.4 mM for lidocaine, respectively (Fig. 1B). Thus, the neurotoxicity in SH‐SY5Y neuronal cells was tetracaine > bupivacaine > ropivacaine > procaine > lidocaine.

### Morphological abnormalities following LAs challenge

Based on the measurements of viability and LD50, the dosages of 200 μM for tetracaine, 900 μM for bupivacaine, 2 mM for ropivacaine, 2.8 mM for procaine and 4 mM for lidocaine were used in all the following experiments. Figure 1C shows the cellular morphology following treatment with LAs. The cells treated with LAs exhibited round and shrunken shapes with the disappearance of neurites. Moreover, most cells treated with LAs lost their cellular integrity compared with untreated control cells.

### LAs increases autophagosome formation

**Figure 2:**
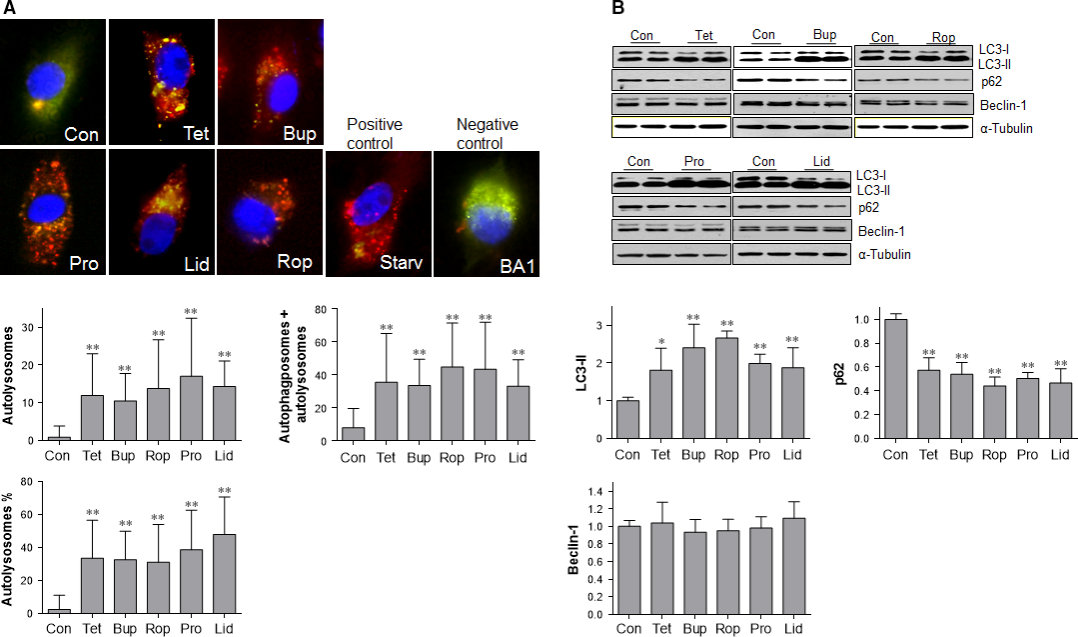
LAs increased autophagic flux in SH‐SY5Y cells. (**A**) Dual fluorescence LC3 assay. Cells were transiently transfected with a dual fluorescent (mRFP‐EGFP) ptfLC3 plasmid. Twenty‐four hours after transfection, cells were stimulated with LAs for 6 hrs. Cells stimulated with serum starvation and bafilomycin A1 served as autophagy‐positive and ‐negative controls, respectively. Yellow puncta represents autophagosomes that did not fuse with lysosomes, whereas red puncta represents autolysosomes that fused of autophagosomes with lysosomes. **P < 0.01 *versus* untreated controls. *n* = 48–188 cells per group. (**B**) LC3‐II, p62 and beclin‐1 levels. Cells were treated with LAs for 6 hrs. Untreated cells served as controls. Cellular extracts were prepared for Western blot with antibodies against LC3, p62 and beclin‐1. The blots against α‐tubulin served as loading controls. **P < 0.01 and *P < 0.05 *versus* untreated controls. *n* = 4–10 per group. Con: control; Tet: tetracaine; Bup: bupivacaine; Rop: ropivacaine; Pro: procaine; Lid: lidocaine; Starv:starvation; BA1: bafilomycin A1.

Autophagosome formation is the first step of autophagy activation. We then examined autophagosome formation using tfLC3 punctuation assay. TfLC3 develops both red and green fluorescence (presents yellow fluorescence after merge) in autophagosomes, whereas only red fluorescence presents in autolysosomes as a result of the quenching of GFP fluorescence by acidic lysosomal environment 27, 29. As shown in Figure 2A, total formed autophagosomes including those fused (red) or not fused (yellow) with lysosomes were increased following treatment with tetracaine (357.6%), bupivacaine (331.3%), ropivacaine (478.5%), procaine (459.5%) and lidocaine (326.8%), respectively, compared with untreated controls (*P* < 0.01). Consistently, LC3‐II generation was significantly up‐regulated following treatment with tetracaine (81.0%), bupivacaine (140.3%), ropivacaine (166.4%), procaine (98.8%) and lidocaine (87.5%), respectively, compared with untreated controls (*P* < 0.01, Fig. 2B). The expression of beclin‐1 was not changed by LAs.

### LAs increases autophagosome clearance

Autophagosome clearance, which begins from the autolysosome formation by fusion of autophagosome with lysosome, is the second step for the complement of autophagic process 27, 30. As shown in Figure 2A, significantly more autolysosomes (red) were observed in the cells treated with tetracaine (1345.8%), bupivacaine (1169.5%), ropivacaine (1571.1%), procaine (1964.0%) and lidocaine (1637.0%), respectively, compared with untreated controls (*P* < 0.01). Similarly, the percentages of autolysosomes in total formed autophagosomes (autophagosome + autolysosomes) were increased by LAs administration. Consistently, the levels of p62, a marker of autophagosome clearance 8, 31, were significantly decreased by treatment with LAs, respectively, compared with untreated controls (*P* < 0.01) (Fig. 2B).

### LAs inhibits mTOR/p70S6K signalling pathway

**Figure 3:**
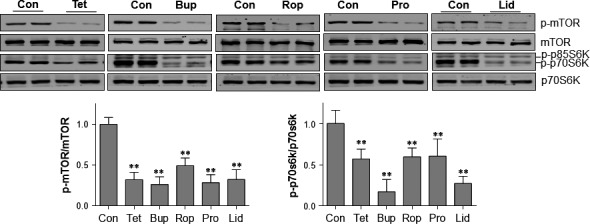
LAs inhibited mTOR/p70S6K activation in SH‐SY5Y cells. Cells were treated with LAs for 6 hrs. Untreated cells served as controls. Cellular extracts were prepared for Western blot with antibodies against p‐mTOR and mTOR, p‐p70S6K and p70S6K. The blots against α‐tubulin served as loading controls. **P < 0.01 *versus* untreated controls. *n* = 4–12 per group. Con: control; Tet: tetracaine; Bup: bupivacaine; Rop: ropivacaine; Pro: procaine; Lid: lidocaine.

mTOR serves as a negative regulator of autophagy activation 14. As shown in Figure 3, the levels of p‐mTOR were decreased by tetracaine (67.9%), bupivacaine (73.8%), ropivacaine (50.8%), procaine (71.6%) and lidocaine (67.5%), respectively, compared with the untreated controls (*P* < 0.01). Consistently, phosphorylation levels of p70S6K, a downstream target of mTOR, were significantly decreased following treatment with LAs, respectively, compared with the untreated controls (*P* < 0.01).

### LAs suppresses tuberin phosphorylation

**Figure 4:**
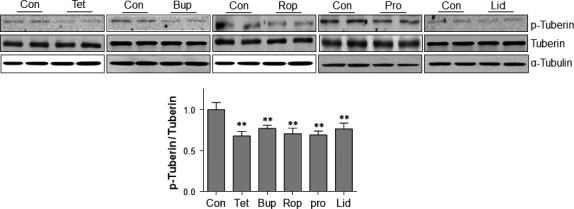
LAs activated tuberin in SH‐SY5Y cells. Cells were treated with LAs for 6 hrs. Untreated cells served as controls. Cellular extracts were prepared for Western blot with antibodies against p‐tuberin and tuberin. The blots against α‐tubulin served as loading controls. **P < 0.01 *versus* untreated controls. *n* = 4–12 per group. Con: control; Tet: tetracaine; Bup: bupivacaine; Rop: ropivacaine; Pro: procaine; Lid: lidocaine.

mTOR activation has been shown to be suppressed by non‐phosphorylated tuberin 16, 17. As shown in Figure 4, the levels of p‐tuberin were decreased by tetracaine (32.0%), bupivacaine (22.9%), ropivacaine (29.4%), procaine (30.8%) and lidocaine (23.5%), respectively, compared with the untreated controls (*P* < 0.01).

### Autophagy inhibition aggravates in the LAs‐provoked neurotoxicity

**Figure 5:**
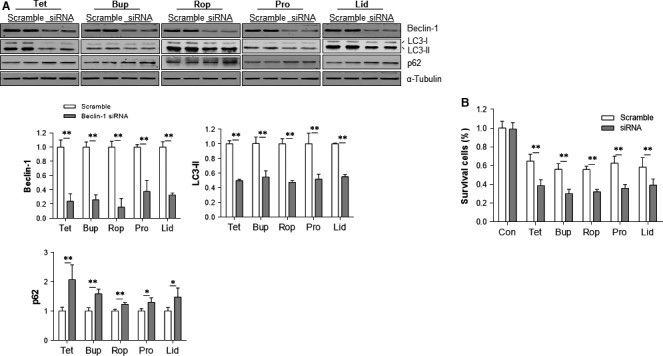
Autophagy inhibition aggravated the LAs‐induced cell death. (**A**) Autophagy inhibition by beclin‐1 knockdown. SH‐SY5Y cells were transfected with beclin‐1 siRNA. The scramble RNA‐transfected cells served as controls. Forty‐eight hours after transfection, cells were exposed to LAs for 6 hrs. Cellular extracts were subsequently prepared for Western blot with antibodies against beclin‐1, LC3 and p62. The blots against α‐tubulin served as loading controls. **P < 0.01 and *P < 0.05. *n* = 4 per group. (**B**) Cell viability. Cells were transfected with beclin‐1 siRNA. The scramble RNA‐transfected cells served as controls. Forty‐eight hours after transfection, cells were exposed to LAs for 24 hrs. Cell viability was evaluated by MTT assay. **P < 0.01. *n* = 5–8 per group. Con: control; Tet: tetracaine; Bup: bupivacaine; Rop: ropivacaine; Pro: procaine; Lid: lidocaine.

To elucidate the roles of autophagy activation in LAs neurotoxicity, we examined the effects of autophagy inhibition on LAs‐induced cells death. Autophagy inhibition was achieved by beclin‐1 knockdown with siRNA transfection. As shown in Figure 5A, beclin‐1 siRNA decreased beclin‐1 expression in the cells treated with all examined LAs (*P* < 0.01). Also, beclin‐1 knockdown significantly decreased LC3‐II contents, whereas increased p62 protein levels in LAs‐treated cells, respectively, compared with the scramble controls (*P* < 0.01, Fig. 5A). Collectively, these data suggest that the LAs‐induced autophagy activation was successfully inhibited by beclin‐1 knockdown.

The effects of autophagy inhibition on LAs‐induced cells death were evaluated by MTT assay. No significant change in viability was detected in the untreated control cells after autophagy inhibition (Fig. 5B). However, autophagy inhibition further decreased viability of the cells treated with tetracaine (35.9%), bupivacaine (41.7%), ropivacaine (34.4%), procaine (50.0%) and lidocaine (50.0%), respectively, compared with the LAs‐treated cells that transfected with scramble RNA (*P* < 0.01).

**Figure 6:**
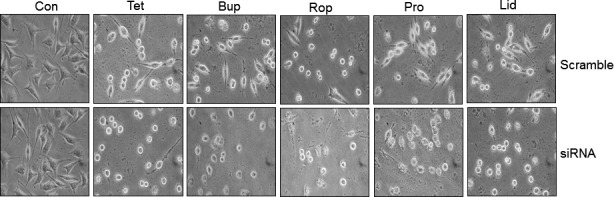
Autophagy inhibition aggravated the LAs‐induced abnormalities in cell morphology. SH‐SY5Y cells were transfected with beclin‐1 siRNA. The scramble RNA‐transfected cells served as controls. Forty‐eight hours after transfection, cells were exposed to LAs for 24 hrs. Cell morphology was observed under a phase‐contrast microscope at a magnification of 400×. Representative images from three independent experiments are shown. Con: control; Tet: tetracaine; Bup: bupivacaine; Rop: ropivacaine; Pro: procaine; Lid: lidocaine.

Figure 6 shows the effects of autophagy inhibition on LAs‐induced cell morphological abnormalities. Autophagy inhibition resulted in severer morphological abnormalities in cells treated with all the examined LAs, respectively, compared with their respective scramble controls.

### LAs increased autophagic markers in primary neurons

**Figure 7:**
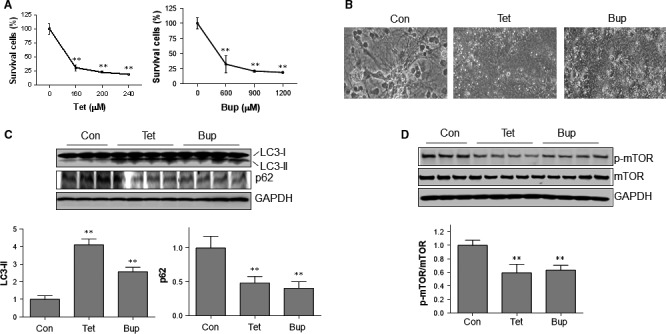
LAs activated autophagic responses in primary cortical neurons. (**A**) Dose‐effects. Neurons were treated tetracaine and bupivacaine for 24 hrs at the indicated concentrations. Cell viability was evaluated with MTT assay. **P < 0.01 *versus* untreated controls (0 μM), *n* = 3/group. (**B**) Cell morphology. Neurons were treated tetracaine (100 μM) and bupivacaine (400 μM) for 24 hrs. Cell morphology was examined with phase‐contrast microscope at a magnification of 400×. The representative images were from three independent experiments. (C) LC3‐II and p62 levels. Primary neurons were challenged with tetracaine (100 μM) and bupivacaine (400 μM) for 6 hrs. Neurons were collected for immunoblotting analysis against indicated antibodies. **P < 0.01 *versus* untreated controls. *n* = 3–4 per group. (**D**) Phosphorylation of mTOR. Primary neurons were challenged with tetracaine (100 μM) and bupivacaine (400 μM) for 6 hrs. Neurons were collected for immunoblotting analysis against indicated antibodies. **P < 0.01 *versus* untreated controls. *n* = 3–4 per group.

To examine whether LAs activate autophagic responses in primary neurons, we treated primary cortical neurons with tetracaine and bupivacaine for 24 hrs at the indicated concentrations. Similar with that in SH‐SY5Y cells, tetracaine and bupivacaine decreased cell viability in a dose‐dependent manner (Fig. 7A). Figure 1B shows the representative morphological abnormalities in the neurons treated with tetracaine (100 μM) and bupivacaine (400 μM) for 24 hrs. LC3‐II formation and p62 protein degradation in primary neurons were examined following treatment with tetracaine (100 μM) and bupivacaine (400 μM) for 6 hrs. As shown in Figure 1C, tetracaine and bupivacaine increased LC3‐II generation by 309.9 and 156.3%, respectively, compared with that in untreated control neurons (*P <* 0.01). By contrast, p62 protein levels were decreased by 51.9 and 59.7%, respectively, compared with that in untreated control cells (*P <* 0.01).

### LAs decreased mTOR phosphorylation in primary neurons

The phosphorylation of mTOR was also examined following treatment with tetracaine (100 μM) and bupivacaine (400 μM) for 6 hrs. As shown in Figure 1D, tetracaine and bupivacaine decreased phosphorylation of mTOR by 40.8 and 36.7%, respectively, compared with that in untreated control neurons (*P <* 0.01).

## Discussion

The significant finding of this study is that autophagic flux was up‐regulated by LAs in 5H‐SY5Y neuronal cells. Suppression of tuberin/mTOR/p70S6K signalling, a negative regulator of autophagy activation, was detected in LAs‐treated cells. Importantly, inhibition of autophagy by beclin‐1 knockdown aggravated the LAs‐provoked neurotoxicity. The results demonstrate for the first time, to the best of our knowledge, that autophagy activation is a general intrinsic protective mechanism against neurotoxicity of both lipid and amide LAs.

Autophagy is a dynamic process including formation and clearance of autophagosomes 8, 12. This flow is defined as autophagic flux 12. During autophagosome formation, a cytosolic form of LC3 (LC3‐I) is conjugated with phosphatidylethanolamine to form LC3‐phosphatidylethanolamine conjugate (LC3‐II), which is recruited to autophagosome membranes. After fused with lysosomes, autophagosomal LC3‐II is degraded together with other components of autophagosomes. Thus, the conversion of LC3 or generation of LC3‐II has been commonly used to examine autophagy activity. However, an increase in LC3‐II contents could be resulted from either enhancement of autophagosome formation or inhibition of autophagosome clearance 7, 12. p62 (also known as SQSTM1) is a critical player for autophagosome formation and is also selectively degraded in an autophagy‐dependent mechanism 7, 8. In this study, we demonstrated that LC3‐II levels were increased, whereas p62 levels were decreased by all the five examined LAs, suggesting that LAs increased autophagic flux in SH‐SY5Y neuronal cells. In supporting this, we observed that autolysosome formation was increased by LAs using dual fluorescence‐based LC3 punctuation assay. Interestingly, we have reported recently that in mouse myoblast C2c12 cells, bupivacaine and lidocaine activated autophagosome formation, whereas blocked autophagic flux as a result of impairment of autophagosome clearance 7. Because it is difficult to use *in vivo* model to evaluate neurotoxicity of LAs, *in vitro* cell culture is widely used in these studies. However, we observed a similar effect of LAs on autophagic responses in primary neurons. Taken together, LAs increased autophagic flux in neuronal cells and can differentially affect autophagic flux in other cell types, in another word, the regulation of autophagy by LAs is cell‐type dependent.

Evidence demonstrates that autophagy is tightly regulated by a series of signalling regulators. Among which, mTOR is known as a central one that negatively regulates autophagy induction 7, 32. As a sensor of nutrients and growth factors, mTOR inhibits autophagy through phosphorylation of multiple autophagy‐related proteins. For example, nutrients and growth factors activate mTOR which in turn leads to inhibition of autophagy by phosphorylation ULK1, ATG13, AMBRA1 and ATG14L, which promote autophagy initiation and autophagosome nucleation 32. mTOR also inhibits lysosomal and autophagy gene expression by phosphorylation and prevention of nuclear localization of the transcription factor EB (TFEB) 32. Proper lysosome function is essential for autophagosome maturation and degradation. Therefore, mTOR participates in the process throughout autophagy induction and completion. In this study, we observed that mTOR was inactivated following LAs administration as demonstrated by decreases in its phosphorylation levels. Also, we observed that the phosphorylation levels of p70SK6, a downstream target of mTOR, were significantly decreased following LAs administration. Moreover, the activity of tuberin (indicated by its non‐phosphorylation levels), a suppressor of mTOR activation, was enhanced following LAs administration. Our data suggest that autophagy activation by LAs may be through removing the inhibitory effects of tuberin/mTOR/p70SK6 on autophagy induction.

Autophagy is a double‐edged sword in the regulation of cell survival 12, 31, 33. In some cases, autophagy may promote cell death. The term ‘autophagic cell death’ or ‘type II cell death’ was coined to differentiate it from apoptotic (type I cell death) or necrotic (type III cell death) 12, 31. As an example, we reported recently that autophagy activation mediates the transition of cardiac hypertrophy to heart failure 33. Moreover, traumatic brain injury‐induced astrocyte death is mediated by autophagy activation 34. On the other hand, lots of studies demonstrate that autophagy also could be cellular protective. Chen *et al*. have shown that increased autophagic flux protects the ethanol‐induced death of SH‐SY5Y neuronal cells 12. To elucidate the roles of activated autophagy in neurotoxicity of LAs in this study, we examined the effects of autophagy inhibition on LAs‐provoked cell injury. We observed that autophagy inhibition with beclin‐1 knockdown exacerbated the LAs‐induced cell injury as reflected by severer morphological abnormalities and decreased cell survival. Collectively, our data indicate that autophagy activation protected neuronal cells from LAs challenge.

In summary, our study demonstrates that LAs up‐regulated autophagic flux through down‐regulation of tuberin/mTOR/p70S6K signalling, and importantly, the autophagy up‐regulation served as a protective mechanism against LAs neurotoxicity. Manipulation of autophagy could be an alternative approach for preventing LAs‐induced neuronal damage.

## Conflict of interest

None declared.
